# Greenhouse gas emissions (GHGE), water footprint and nitrogen loss associated with food consumption among adults: findings from the updated LEBANese natiONal food consumption survey (LEBANON-FCS)

**DOI:** 10.1186/s40795-025-01004-6

**Published:** 2025-01-27

**Authors:** Maha Hoteit, Maroun Khattar, Esraa Antar, Dana Malli, The Adults-Lebanon-FCS Group, Zahraa Fadlallah, Razan Khadra, Mohamad Chahine, Omasyarifa Binti Jamal Poh, Nikolaos Tzenios, Elham Al Manasfi, Abdulrahman Chahine

**Affiliations:** 1 Food Sciences Unit, National Council for Scientific Research of Lebanon (CNRS-L), Beirut, Lebanon; 2 PHENOL Research Group (Public Health Nutrition Program-Lebanon), Faculty of Public Health, Lebanese University, Beirut, 6573 Lebanon

**Keywords:** Adult, Food consumption, Sustainability, Environmental footprint, Lebanon

## Abstract

**Background:**

Lebanon is grappling with numerous environmental challenges, including water scarcity, landfill waste, deforestation, and rising air pollution. Food choices significantly influence global greenhouse gas emissions and environmental impacts, making it crucial to evaluate the environmental footprints (EFPs) of Lebanon’s current dietary habits. This study aimed to assess food consumption patterns and their EFPs among a nationally representative sample of Lebanese adults.

**Methods:**

A cross-sectional survey was conducted from May to September 2022, involving 444 Lebanese adults aged 18 to 64 years. The sample was representative, and participants were distributed across the eight Lebanese governorates. Sociodemographic and medical data were collected via a questionnaire, food consumption was assessed through a validated Food Frequency Questionnaire (FFQ) and two non-consecutive 24-hour recalls, and anthropometric measurements were also taken. EFPs were derived from databases and repositories.

**Results:**

The typical EFPs of an average Lebanese adult included water usage of 2,862.39 ± 1,617.88 L/day, greenhouse gas emissions of 4.43 ± 2.29 kg CO2-eq/day, and nitrogen use of 12.72 ± 6.76 g/day. Animal products were the primary contributors to greenhouse gas emissions, while vegetable products had the highest water footprint and nitrogen loss impact on the environment. Grains and cereals, the most consumed food category, significantly influenced the water footprint and nitrogen loss. Additionally, meat consumption notably drove greenhouse gas emissions.

**Conclusion:**

Lebanon must address its environmental challenges and the impact of dietary choices on greenhouse gas emissions and EFPs. By evaluating and understanding the environmental consequences of current dietary patterns, Lebanon can take proactive steps towards promoting sustainable food practices and mitigating environmental degradation.

**Supplementary Information:**

The online version contains supplementary material available at 10.1186/s40795-025-01004-6.

## Background

Based on the ‘United Nations Environment Programme’ (UNEP), the world is facing a climate emergency, and warming is expected to exceed 2.9 °C if no efficient actions are taken to decrease greenhouse gas emissions (GHGE) dramatically [1]. For instance, in 2023, the world experienced a new record in GHGE, and reports showed that most of the 2030 ‘Sustainable Development Goals’ are off track, especially SDG13 that calls for climate action [2]. One of the major contributors to this GHGE is households, as it is reported that their consumption contributes to 60% of the global GHGE [3]. This highlights the importance of making people’s dietary patterns more environmentally sustainable by promoting “Environmentally Sustainable Food Consumption” (ESFC). ESFC can be defined as using goods and services needed to fulfill basic needs and improve the quality of life without jeopardizing the ability of the future generations to fulfill their own needs [4]. This is achieved by minimizing the reliance on natural resources, materials that are toxic, waste, and pollutants’ emissions over the lifecycle [4]. One example of ESFC is the double pyramid developed by the Barilla Center for Food & Nutrition [5] that encourages the consumption of plant-based food due to their low environmental footprint and adequate nutritional value while minimizing the consumption of red meat and sweets that have a high environmental footprint and are linked to the development of nutrition-related non-communicable diseases (NR-NCDs). However, encouraging sustainable consumption is faced by people’s food preferences and cultural habits, which play a role in shaping a person’s food habits and dietary pattern. In short, dietary choices are subject to affective, environmental, cognitive, and social influences [6], and all these should be taken into consideration when planning to encourage ESFC.

Despite the importance of such a topic, data on the environmental footprint of food consumption in Lebanon is scarce, and the data available represents the consumption patterns of the year 2009 [7], which changed a lot in the recent years, as evidenced by a recent study that compared the adults’ consumption patterns of the year 2022 to those of 2009 [8]. Thus, it is important to assess the environmental footprint of the current consumption to gain a recent insight into the impact of this consumption on the country’s scarce environmental assets. Such data is critical to shaping future policies aiming at promoting ESFC, which is critical in a country that is encountering a wide range of environmental challenges (water shortages, accumulation of waste in landfills, destruction of forests, and a surge in air pollution…), as reported by the ‘United Nations Development Programme’ (UNDP) in 2021 [9].

## Methods

### Study design and eligibility criteria

This was a cross-sectional study that was conducted over a period of 5 months between May and September 2022 on a nationally representative sample of Lebanese adults. To be eligible, a participant had to be Lebanese and aged between 18 and 64 years old.

### Sampling method and recruitment process

For the sample to be nationally representative, a minimum number of 400 participants was required. The sample size was calculated based on the population estimates from 2018 to 2019 using the following formula:

$$\mathrm n=\left[\mathrm p\;\left(1-\mathrm p\right)\right]\times\left[\left({\mathrm Z}_{\propto/2}\right)^2/\left(\mathrm e\right)^2\right]$$

where ‘n’ refers to the sample size; ‘Z_∝/2_’ refers to the standard error’s reliability coefficient at a 5% level of significance and is equal to 1.96; ‘p’ denotes the probability of adults (18–64 y) who were not capable of taking precautions regarding the diseases (50%); and ‘e’ represents the standard error’s tolerated level (5%), as stated by Hosmer and Lemeshow. To ensure the adequacy of our sample size and the statistical robustness of our study, a power analysis was conducted. Given the distribution of participants (184 males and 265 females), a post-hoc power analysis was performed for the primary outcomes of interest. Assuming a medium effect size (Cohen’s d = 0.5), an alpha level of 0.05, and a desired power of 0.80, the analysis confirmed that the sample size of 444 participants provided sufficient statistical power to detect meaningful differences or associations between the stratified groups. This calculation was based on a two-sample t-test for comparing continuous outcomes between males and females. To estimate the impact of the design effect, we used the standard formula:

$$\mathrm{Design}\;\mathrm{Effect}(\mathrm{DEFF})=1+\left(\mathrm m-1\right)\mathrm\rho\backslash\mathrm{text}\;\left\{\mathrm{Design}\;\mathrm{Effect}\right\}\;\left(\mathrm{DEFF}\right)\;=1+\left(\mathrm m-1\right)\backslash\mathrm{rho}\;\mathrm{Design}\;\mathrm{Effect}(\mathrm{DEFF})=1+(\mathrm m-1)\mathrm\rho$$

Where mmm is the average cluster size, and ρ\rhoρ is the intra-cluster correlation coefficient (ICC). Based on assumptions of a small ICC (common in dietary studies) and the distribution of participants across clusters, the DEFF was calculated to be minimal. The effective sample size was adjusted accordingly, and the results still retained sufficient power for the primary outcomes of interest [10].

The stratified cluster sampling method and the inclusion of diverse recruitment channels ensured proportional representation across the eight Lebanese governorates and both genders. These methods enhanced the generalizability of the findings and mitigated potential biases, while limiting participation to one individual per household further strengthened the representativeness of the sample.

**Fig. 1:**
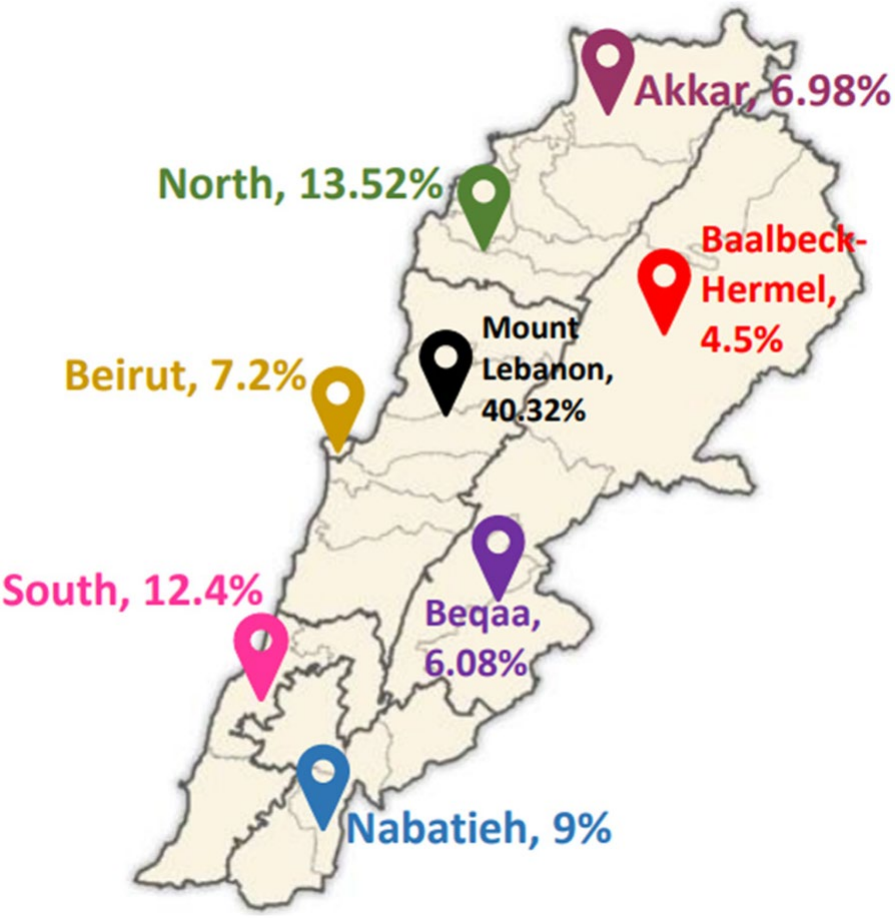
Distribution of study participants across the 8 Lebanese governorates [11]

Overall, 449 participants (184 males and 265 females) from the 8 Lebanese governorates were included. The sampling technique was a combination of stratified cluster sampling, with the stratified groups being the two genders and the clusters being the Lebanese governorates. The process of recruiting participants involved various channels, including volunteers, charitable organizations, first-aid and medical centers, so a broad range of participants could be reached. Participants recruited in this stage were then encouraged to invite other individuals within their outreach to participate. This allowed us to reach participants that were difficult to reach physically due to budget constraints and timing. The individuals willing to participate were informed about the study nature and then assessed for eligibility. Eligible participants provided an electronic written consent indicating their willingness to participate in the study. One individual per Lebanese household was allowed to participate in the study so that a wide representation of households could be guaranteed. Overall, the study involved 444 participants, as 5 participants were excluded after checking for missing data, errors, and outliers. Participant distribution across the 8 governorates is shown in Fig. 1.

## Data collection

### Sociodemographic questionnaire

Participants were interviewed, and data were collected using a pretested questionnaire that asked about the demographic, socioeconomic, and medical characteristics of the participants. For instance, weight, height, age, gender, marital status, education level, and current occupation were among the data collected. Additionally, data about household size and number of rooms was collected in order to calculate the crowding index (CI), which is a representation of a household’s socioeconomic status [12].

### Food frequency questionnaire

In this phase, a 30-min interview was conducted by trained dietitians to collect data about the dietary intake of the study participants. This was done by administering a valid semi-quantitative food frequency questionnaire (FFQ) composed of 157 food items and two non-consecutive 24-h recalls [13]. Visual aids and explanations about portion sizes were provided to participants to help them remember what they ate and estimate the portions as accurately as possible. Consuming servings were recorded as monthly, weekly, and daily.

### Anthropometric measurements

In this phase, the weight and height of every participant were measured three times to increase accuracy, and the Body Mass Index (BMI) was calculated after getting the average of the three readings.

### Environmental footprints derivation

The corresponding carbon and water footprints of every food item included in the FFQ were extracted from different sources, including the Barilla Center for Food & Nutrition Double Pyramid [5] and the carbon and water footprint dataset of food commodities developed by Petersson et al. [14]. As for the nitrogen footprint, due to a lack of data in the country about food production and other factors used when calculating the nitrogen footprint, we only calculated the nitrogen content of the protein consumed. Physiologically, unless being an athlete aiming to gain muscle mass, nitrogen coming from protein consumed is excreted, and we based our work on this assumption as it is used elsewhere [15, 16]. The nitrogen content of the food consumed by study participants was calculated by dividing every food item’s protein content by the corresponding nitrogen factor extracted from the USDA Agricultural Research website [17]. After extracting the corresponding EFPs in every kilogram of item consumed, each item’s consumption in kg/d was multiplied by its corresponding EFP to calculate its contribution to the footprint in the study sample. For composite dishes (salads, traditional dishes and sweets, burger, and shawarma sandwiches), standardized recipes were obtained, and each ingredient’s EFP was multiplied by its percent contribution to the recipe to get the overall EFPs of the food/dish as indicated by the Barilla Center for Food and Nutrition Double Pyramid [5].

### Drafting the double pyramid simulation based on current consumption

The Barilla Center for Food and Nutrition Double Pyramid [5] was adopted to compare the current consumption with its recommendations, and we classified our food items into food groups according to this pyramid’s classification to allow comparison. For instance, based on this pyramid, cheese was separated from milk and yogurt as it has higher EFPs compared to them, so we calculated the EFPs of cheese separately in this study. This helped us in assessing if the current consumption of our sample is in line with the recommendations or if a deviation in consumption occurred, and where it exactly occurred to come up with suggestions that are beneficial for both the health of the Lebanese population and Lebanon’s environmental assets.

### Data management and statistical analysis

Data was managed using Microsoft Excel 2016. The amount of food consumed, recorded as monthly, weekly, or daily, was unified to ‘daily’ consumption by dividing monthly consumption by 30 and weekly consumption by 7. After getting the amount of food consumed daily (grams per day; g/d) and extracting the corresponding environmental footprints, the mean and standard deviation (SD) of the continuous variables and frequencies (N) and percentages (%) of the categorical variables were calculated using The Statistical Package for the Social Sciences (SPSS; Version 25.0, IBM Corp: Armonk, NY, USA).

## Results

### Participants characteristics

Participants’ demographic and socioeconomic characteristics are shown in Table 1. Their health characteristics are shown in Table 2. Of the participants, 58.8% were females. Most of the participants were from the Mount Lebanon governorate (40.32%), and almost half were married (50.23%). Based on the crowding index, we can imply that the majority of the households have a low socioeconomic status, as the majority (62.84%) are crowded. About half of the sample reported being unemployed (49%), and more than one-quarter of the sample (27.7%) experienced a decline in their salary due to the economic crisis. The majority of participants were overweight or obese based on their BMI (61.9%), and the mean BMI was 27.2 kg/m^2^ for male participants and 26.9 kg/m^2^ for female participants. The majority of participants (75%) reported having no chronic diseases. As for the participants who reported having one or more chronic diseases, anemia and hypertension were the most prevalent.

**Table 1:** Demographic and socioeconomic characteristics of the participants, overall and by gender

		Overall (*n* = 444)	Male (*n* = 183)	Female (*n* = 261)		*P*-value
		*N* (%)	*N* (%)	*N* (%)		
Age Category	18 Years	20 (4.5%)	5 (25%)	15 (75%)		0.167
	19–30 Years	187 (42.1%)	78 (41.7%)	109 (58.3%)		
	31–50 Years	174 (39.2%)	79 (45.4%)	95 (54.6%)		
	51–64 Years	63 (14.2%)	21 (33.3%)	42 (66.7%)		
Residency (Governorate)	Akkar	31 (6.98%)	11 (35.5%)	20 (64.5%)		0.006*
	Mount Lebanon	179 (40.32%)	73 (40.8%)	106 (59.2%)		
	Beqaa	27 (6.08%)	5 (18.5%)	22 (81.5%)		
	North Lebanon	60 (13.52%)	33 (55%)	27 (45%)		
	Baalbek-Hermel	20 (4.5%)	10 (50%)	10 (50%)		
	South Lebanon	55 (12.4%)	30 (54.5%)	25 (45.5%)		
	Beirut	32 (7.2%)	10 (31.2%)	22 (68.8%)		
	Nabatiyeh	40 (9%)	11 (27.5%)	29 (72.5%)		
Marital Status	Single	202 (45.5%)	81 (40.1%)	121 (59.9%)		0.139
	Married	223 (50.23%)	97 (43.5%)	126 (56.5%)		
	Widowed	7 (1.57%)	0 (0.0%)	7 (100%)		
	Divorced	12 (2.7%)	5 (41.7%)	7 (58.3%)		
Crowding index	No crowding	165 (37.16%)	79 (47.9%)	86 (52.1%)		0.028*
	Crowding	279 (62.84%)	104 (37.3%)	175 (62.7%)		
Number of children	0	217 (48.87%)	87 (40.1%)	130 (59.9%)		0.124
	1–3	159 (35.81%)	74 (46.5%)	85 (53.5%)		
	> 3	68 (15.32%)	22 (32.4%)	46 (67.6%)		
Education level	Illiterate	3 (0.68%)	1 (33.3%)	2 (66.7%)		0.961
	School	175 (39.41%)	72 (41.1%)	103 (58.9%)		
	University	266 (59.91%)	110 (41.4%)	156 (58.6%)		
Current Occupation	Unemployed	218 (49.1%)	40 (18.3%)	178 (81.7%)		< 0.001*
	Employed	226 (50.9%)	143 (63.3%)	83 (36.7%)		
Monthly salary changes after economic crisis	No Impact	129 (29%)	63 (48.8%)	66 (51.2%)		< 0.001*
	Decline in Salary	123 (27.7%)	51 (41.5%)	72 (58.5%)		
	Increase in Salary	70 (15.8%)	42 (60%)	28 (40%)		
	Already have no Salary	122 (27.5%)	27 (22.1%)	95 (77.9%)		
Household Monthly Income	None	39 (8.78%)	17 (43.6%)	22 (56.4%)		< 0.001*
	Less than 1.5 million LBP	58 (13.1%)	15 (25.9%)	43 (74.1%)		
	>=1.5 million LBP	211 (47.5%)	79 (37.4%)	132 (62.6%)		
	<=300 USD	92 (20.72%)	41 (44.6%)	51 (55.4%)		
	More than 300 USD	44 (9.9%)	31 (70.5%)	13 (29.5%)		

*P-value < 0.05 is significant

**Table 2:** Health characteristics of the study population, overall and by gender

		Overall (*n* = 444)		Male (*n* = 183)		Female (*n* = 261)		
		Mean	SD	Mean	SD	Mean	SD	*P*-value
Weight (kg)		73.8	17.1	81.8	16.6	68.3	15.1	< 0.001*
Height (cm)		165.3	9.4	173.5	7	159.5	5.9	< 0.001*
Body mass index (kg/m^2^)		27	5.8	27.2	5.3	26.9	6.1	0.626
		N	%	N	%	N	%	
BMI classification	Underweight	19	4.3	4	21.1	15	78.9	0.084
	Normal	150	33.8	57	38	93	62	
	Overweight & Obese	275	61.9	122	44.4	153	55.6	
Disease status	No disease	333	75	150	45	183	55	0.005*
	Having disease	111	25	33	29.7	78	70.3	
Disease type	Cardiovascular disease	14	12.6	5	15.2	9	11.5	0.004*
	Diabetes	3	2.7	1	3	2	2.6	
	Hypertension	34	30.6	16	48.5	18	23.1	
	Kidney disease	4	3.6	2	6.1	2	2.6	
	Liver disease	1	0.9	1	3	0	0	
	Osteoporosis	14	12.6	1	3	13	16.7	
	Asthma/Respiratory	12	10.8	2	6.1	10	12.8	
	diseases	36	32.4	5	15.2	31	39.7	
	Anemia	32	28.8	6	18.2	26	33.3	
	Others ^a^							

^*^P-value < 0.05 is significant

^a^ Includes other self-reported diseases: (1) Allergies (seasonal, food, dust, skin); (2) Vertebral column problems; (3) Sarcoidosis; (4) Migraine; (5) Thyroid disease; (6) Gastrointestinal problems; (7) Psychological conditions; (8) Neurological conditions; (9) Hypovitaminosis D; (10) Hypocalcemia; (11) Iron deficiency; (12) Urinary tract infection; (13) Hypercholesterolemia; (14) Raynaud’s syndrome; (15) Varicose veins; (16) Autoimmune diseases; (17) Cancer; (18) Thrombosis; (19) Polycystic ovarian syndrome

## Environmental impact of food consumed by study participants

### Food consumed by the study participants

Table 3 summarizes the dietary characteristics of food consumed by study participants. Mean dietary intake was 2237.49 ± 1214.38 kcal/day. Carbohydrates, proteins, and fats contributed to 56.6%, 14.29%, and 30.44% of energy intake, respectively. Table S1 shows the food items included in every food group. Grains and cereals (317.18 g/d) were the most consumed food group, followed by fruits and fresh fruit juices (292.33 g/d), non-starchy and starchy vegetables (256.97 g/d), and dairy products (184.1 g/d). Compared to the healthy diets’ recommendations (USDA, Mediterranean, and EAT-Lancet diets), the pattern is characterized by a high consumption of red meat, sweets and added sugars, and saturated fats, and a low consumption of seafood, nuts and seeds. In addition, the quantity of grains and cereals (g/d) consumed is within recommendations; however, 92% of the grains and cereals consumed are refined, which exceeds the recommendations by far (at least 50% should be whole grains) [18–21].

**Table 3:** Dietary characteristics of food consumed by study participants

Food Groups (g/d)	Dietary Intake Characteristics
	Total Population (*n* = 444)
Bread, Cereals and Grains	317.18 ± 134.85
Legumes	66.85 ± 54.77
Nuts & Seeds	5 ± 12.46
Starchy Vegetables	50.48 ± 43.48
Vegetables	206.49 ± 117.79
Dairy Products	184.5 ± 164.72
**Meat & Meat Products, Poultry, Fish, Eggs**	
Red Meat	41.34 ± 48.39
Processed Meat	3.11 ± 5.32
Poultry	34.21 ± 42.34
Fish	11.39 ± 21.02
Eggs	22.23 ± 32.6
**Fruits, Total**	
Fruits	254.33 ± 284.95
Fresh Juices (100% fruit juices) ^a^	38 ± 75.5
**Sweets & Added Sugars**	
Sweets	65.36 ± 114.08
Added Sugars, Jams, Honey, Molasses	18.97 ± 22.15
Added Fats & Oils	11.51 ± 11.68
Herbs and Spices	47.87 ± 44.1
Hot Beverages ^a^	546.83 ± 427.36
Drinking Water ^a^	1440.48 ± 843.19
Non-alcoholic Beverages ^a^	70.63 ± 120.94
Alcoholic Beverages ^a^	0.6 ± 5.9
**Energy and Macronutrient Content**	
Energy Intake (kcal)	2237.49 ± 1214.38
Carbohydrates (g, % of total energy)	316.65 ± 163.7, 56.6%
Proteins (g, % of total energy)	79.99 ± 48.65, 14.29%
Fat (g, % of total energy)	75.68 ± 50.9, 30.44%

Values are Mean ± Standard deviation

^a^ Beverages presented in ml/day

### Environmental footprints of food consumed by the study participants

Table S2 shows the EFPs used for food items in our study. Table 4 shows the environmental footprints (EFPs) of the food consumed by our study population. On average, the dietary pattern followed by the study population has the following EFPs (mean ± SD): Water use: 2,862.39 ± 1,617.88 L/day; GHGE: 4.43 ± 2.29 kg CO_2_-eq/d; Nitrogen: 12.72 ± 6.76 g/d. In 1000 kcal consumed, the dietary pattern followed would have the following EFPs: Water use: 1,436.73 ± 865.95 L/day; GHGE: 2.2 ± 1.34 kg CO_2_-eq/d; Nitrogen: 6.39 ± 3.93 g/d.

**Table 4:** Environmental footprints of food consumed by the study participants

	Total Population (*n* = 444)
	Mean ± SD
Water Use (L)	2,862.39 ± 1,617.88
Water Use/1000 kcal	1,436.73 ± 865.95
GHGE (as kg CO_2_-eq)	4.43 ± 2.29
GHGE (as kg CO_2_-eq)/1000 kcal	2.2 ± 1.34
Nitrogen (g)	12.72 ± 6.76
Nitrogen (g)/1000 kcal	6.39 ± 3.93

Table 5 shows the EFPs of the food consumed by food group. Meat was the most significant contributor to GHGE (1.16 kg CO_2_-eq/d), followed by “grains and cereals” (0.8 kg CO_2_-eq/d), “vegetables” (0.61 kg CO_2_-eq/d), and “hot beverages” (0.44 kg CO_2_-eq/d). Overall, animal products contributed to the highest (1.95 kg CO_2_-eq/d) GHGE in our study sample. For water footprint, “grains and cereals” (604.79 L) contributed to the highest footprint, followed by meat (522.25 L) and “sweets & added sugars” (319.6 L). Overall, vegetable products contributed to the most water use in our study sample (1313.63 L). As for nitrogen content in the food consumed, “grains and cereals” (4.04 g) contributed to the highest loss to the environment, followed by meat (1.83 g), poultry (1.39 g), and “milk, yogurt, labneh” (1.02 g).

**Table 5:** Environmental footprint of food consumed by study participants, per food group

Food Group	GHGE (as kg CO_2_-eq)	Nitrogen Content (g)	Water Footprint (L)
**Animal Products**	**1.95**	**5.93**	**1,022.93**
Meat (Red, Processed)	1.16	1.83	522.25
Poultry	0.16	1.39	132.21
Fish	0.05	0.45	29.2
Eggs	0.11	0.44	73.39
Milk, Yogurt, Labneh	0.28	1.02	157.37
Cheese	0.19	0.8	108.51
**Vegetable Products**	**1.64**	**6.2**	**1,313.63**
Grains & Cereals	0.80	4.04	604.79
Vegetables (Starchy & Non-starchy)	0.61	0.8	151.15
Fruits	0.12	0.39	184
Legumes	0.05	0.74	171.72
Olive Oil	0.03	0	118.9
Olives, Nuts & Seeds	0.03	0.23	83.07
**Others**	**0.84**	**0.59**	**525.83**
Added Fats & Oils	0.02	0	36.79
Sweets, Snacks & Added Sugars	0.23	0.55	319.6
Sugar Sweetened Beverages	0.15	0.04	104.57
Hot Beverages	0.44	0	64.87

**Fig. 2:**
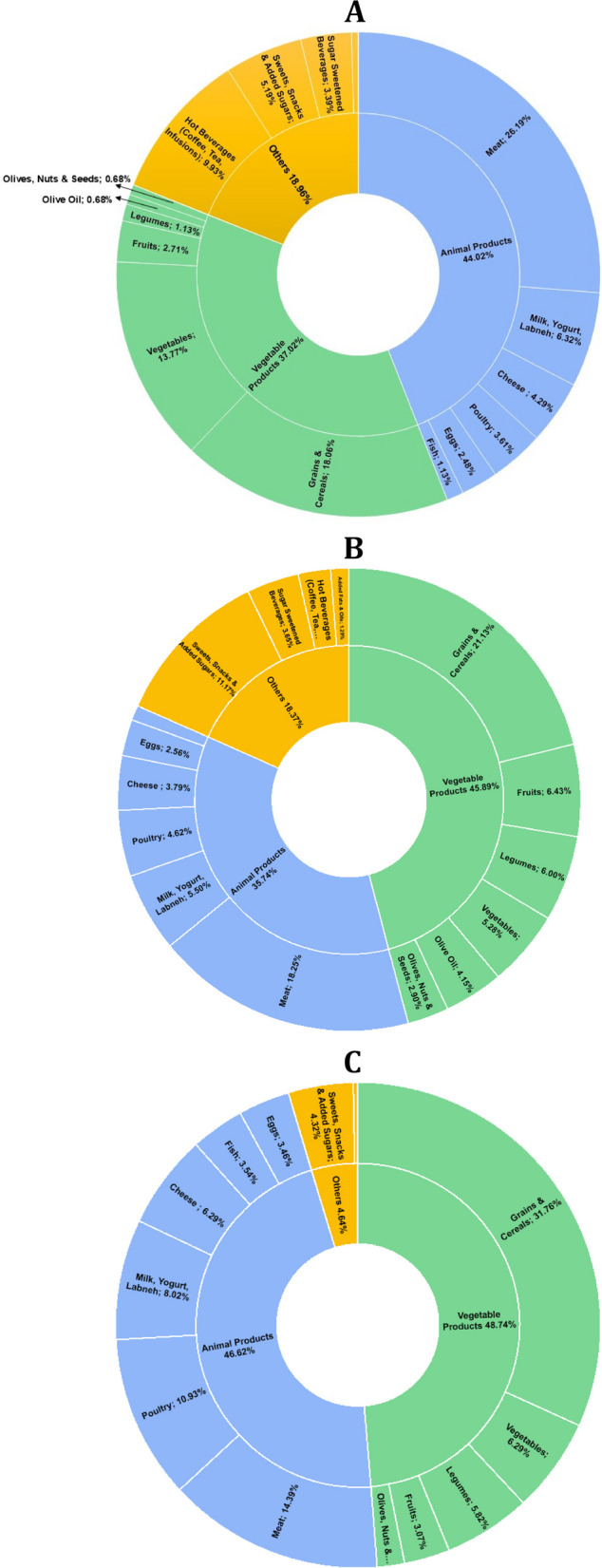
Percent contribution of every food group to the EFPs (**A** GHGE, **B** Water, **C** Nitrogen)

The percentage contribution of every food group and item to the EFPs is shown in Fig. 2. For GHGE, animal products were the main contributors (44.02%), with meat alone contributing to 26.19% of the total GHGEs and more than 50% of the GHGEs coming from the consumption of animal products. Fish (1.17%) contributed the least to the GHGE coming from animal products in our study sample. “Grains and cereals” contributed to the highest GHGEs coming from vegetable products (18.06%), followed by vegetables (13.77%), while legumes (1.13%), olive oil (0.68%), and ‘olives, nuts and seeds’ (0.68%) contributed to the least GHGEs (see Fig. 2A). For water use, vegetable products contributed to the highest footprint in our study sample (45.89%), among which “grains and cereals” contributed to 21.13% of the overall water footprint (46% of the water footprint coming from vegetable products), while “olives, nuts & seeds” (2.9%), olive oil (4.15%), and vegetables (5.28%) contributed the least. Overall, “grains and cereals” (21.13%) contributed to the highest water footprint in our sample, followed by meat (18.25%) and ‘sweets, snacks, and added sugars’ (11.17%). Fish (1.02%) contributed to the least water footprint in our study sample (see Fig. 2B). As for nitrogen content of food consumed, vegetable products contributed the most (48.74%), with grains and cereals contributing alone to 31.76% (65% of the nitrogen in this group). Animal products contributed 46.62% of the nitrogen, with meat contributing the most (14.39%), followed by poultry (10.93%). Overall, grains and cereals contributed the most to the nitrogen content (31.76%), followed by meat (14.39%) and poultry (10.93%) (see Fig. 2C).

### Food-environmental pyramid and current consumption

**Fig. 3:**
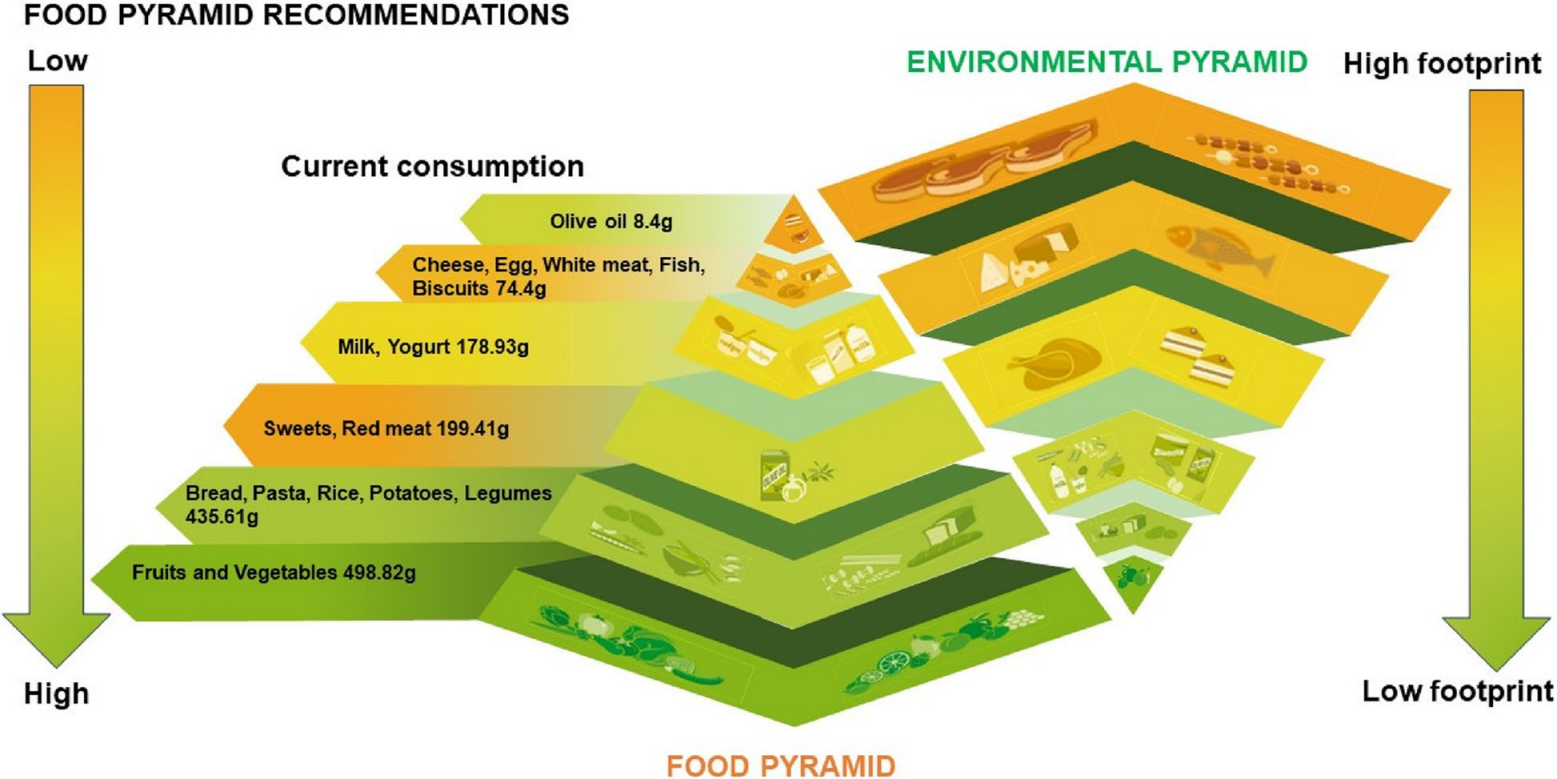
Double pyramid and current consumption (Colors reflect the recommended frequency of consumption by the Double Pyramid) [5]

A comparison between the current consumption and the Double Pyramid recommendations is shown in Fig. 3. The Double Pyramid classifies food into categories, taking into account their environmental footprints. At the bottom of the ‘environmental pyramid’ are foods that have the highest EFPs and must thus be less frequently consumed, and that’s why they are at the top of the ‘consumption pyramid’ (red shade of color). In contrast, foods that have the lowest EFPs are at the top of the ‘environmental pyramid’ and thus must be consumed more frequently (placed at the bottom of the ‘consumption pyramid’) (green shade of color). A deviation in the consumption of red meat, sweets, and olives occurred in our sample. For instance, red meat and sweets are recommended to be consumed in the least amounts due to their high EFPs and their role in the development of NR-NCDs if consumed in large amounts, while olive oil is recommended to be consumed in larger amounts due to its health benefits and low EFPs. However, in our sample, red meat and sweets were the 3rd most consumed food group instead of being the least, and olive oil was the least consumed food group instead of being the 3rd most consumed.

## Discussion

Data available in the country regarding the EFPs of Lebanese adults’ dietary patterns belong to the pattern of the year 2009 [7]. Although important, it was crucial to assess the EFPs of the current dietary patterns due to the shift that occurred because of the many factors (COVID-19, the Beirut blast, and the economic crisis) that affected the country since then. This study presents updated data on this topic, which is valuable in a country struggling economically and environmentally. Compared to the previous data, the overall GHGE slightly increased from 4.06 kg CO_2−_eq/d to 4.43 kg CO_2−_eq/d, and the overall water footprint increased from 2,571.62 L/d to 2,862.39 L/d. To allow a better comparison, EFPs in 1000 kcal were used. The previous EFPs (year 2009) reported in 1000 kcal were 1.53 kg CO_2−_eq/1000 kcal and 951.68 L/1000 kcal for GHGE and water footprint, respectively [7]. Based on the current dietary patterns, the GHGE increased to 2.2 kg CO_2−_eq/1000 kcal, and the water footprint increased to 1,436.73 L/1000 kcal. This can be explained by the increased consumption of fruits, dairy products, chips, salty crackers, sweets, added sugars, and hot beverages compared to the year 2009, faced by a decrease in the consumption of sugar-sweetened beverages and alcohol only. To compare our findings with other countries, EFPs in 2500 kcal/d were calculated and were as follows: Mean ± SD: GHGE: 5.5 ± 3.93 kg CO_2−_eq; water footprint: 3,591.83 ± 2,164.88 L/d; nitrogen content: 15.98 ± 9.82 g.

For water use, the average use per person was higher than the global average (2,799 L/d) [22]. Higher estimates were obtained in North America (3,999 L/d) [23], while lower estimates were obtained in Tunisia (3,292 L/d) [24] and Finland (2,377 L/d) [23]. Almost similar estimates were obtained in Italy (3,469 L/d) [23]. In our study, grains and cereals (21.13%) were the most contributing to the water footprint, followed by meat (18.25%). In Italy [23], cereals and bakery products were the most contributors to the total water footprint, which aligns with our findings. In Tunisia [24], meat was the main contributor to the water footprint, followed by cereals, which are the two most contributing groups in our study. The findings of our study showed that the current consumption’s water footprint exceeded the global average, which is alarming in a country facing many environmental problems such as air pollution, waste accumulation, and water shortages [9]. For instance, Lebanon’s renewable water resources are currently less than 1000m^3^/capita/year (2740 L/d), which is the threshold that defines “water stress” [9].

For GHGEs associated with the current dietary pattern in Lebanon, an average of 5.5 kg CO_2−_eq is emitted per day. Higher estimates were obtained in the United Kingdom (7.4 kg CO_2−_eq/d) and Australia (19.5 kg CO_2−_eq/d) [25, 26], while lower estimates are found in the United States (3.55 kg CO_2−_eq/d) [27] and France (4.8 kg CO_2−_eq/d) [28]. Animal products were the main contributors to GHGEs in our study. Meat was the most contributor to the GHGEs, followed by the ‘grains and cereals’ group. Meat and meat alternatives contributed to the highest GHGEs in Australia (33.9%) and the UK (32%) [25, 26], which aligns with our findings (26.45%). Meat and meat alternatives were also the main contributors to GHGEs in France [28]. The current findings are considered alarming, as a previous report stated that GHGEs in the country increased by 129% between 1994 and 2015 [9].

In addition to the GHGEs and water footprint, this study is the first in Lebanon and the Arab Region to assess the nitrogen content of food consumed, which is eventually lost to the environment. Assessing nitrogen lost to the environment is crucial because nitrogen is the most prolific gas in the atmosphere [29]. For instance, nitrogen lost to the environment can cause pollution that harms the environment in many ways. Based on the UNEP, nitrous oxide (N_2_O) is way more powerful than carbon dioxide (CO_2_) and methane (CH4) as a greenhouse gas and is believed to be the greatest human-made threat to the ozone layer [29]. In Ukraine [30], the consumption of plant-based food led to a higher nitrogen footprint (2.35 kg/year) compared to animal food (1.79 kg/year), which aligns with our findings (2.26 kg/year and 2.16 kg/year for plant-based food and animal-based food, respectively). Overall, the contribution of these two groups to the nitrogen footprint was 4.14 kg/year in Ukraine and 4.42 kg/year in our study, which is almost similar. In addition, cereals were the main contributors to the plant-based nitrogen footprint, similarly to our findings [30]. Contrasting our findings, milk was the main contributor from the animal products in Ukraine [30], while meat was the main contributor in our study.

Compared to environmental constraints based on data from 152 countries [31], the water footprint in our study sample represents 364% of the limit (786 L/cap/day), GHGEs represent 237% of the limit (1,866 g CO2-eq/cap/day), and the amount of nitrogen lost to the environment represents 46% of the limit set (27.4 g Nitrogen/cap/day). It is important to note that the nitrogen footprint in our study represents only consumption, which is alarming as it alone exceeded 45% of the limit.

The current findings, along with the findings of the recent study that assessed the nutritional value of the dietary pattern of Lebanese adults [8], show that this pattern is lacking essential vitamins and minerals and high in EFPs. Shifting towards healthier and more sustainable diets is therefore crucial to improve both the health of the population and the environmental assets. Such a positive shift can be following the Barilla Center for Food & Nutrition Double Pyramid (Food-Environmental Pyramid) [5], which shows that food that are recommended to be consumed in higher proportions have lower environmental footprints. Cheese in this double pyramid is separated from other dairy products as it has higher EFPs compared to milk and yogurt. Based on the Double Pyramid, fruits have the lowest EFPs, followed by vegetables and cereals, and thus these groups are recommended to be consumed in higher amounts. For better health benefits, grains and cereals are preferred to be whole grains, which is not the case in our study. As can be seen in Fig. 3, the deviation from the Double Pyramid in our sample occurred with sweets and red meat being the 3rd most consumed food items (instead of being the least) and olive oil the least consumed item (instead of being the 3rd ). As such, a decrease in the consumption of sweets and red meat in our sample and an increase in the consumption of olive oil would benefit both the health of our population and the environment. For instance, red meat and sweets have high EFPs and are low in essential vitamins and minerals and are linked to many chronic diseases, while olive oil has lower EFPs and is healthier as it offers many health benefits by being high in monounsaturated fatty acids, which are healthy fats that lower the risk of cardiovascular diseases and inflammation [32]. Overall, the consumption of more plant-based food and lowering the consumption of animal-based food is recommended as it would benefit both people and the environment. Besides the Double Pyramid, the Mediterranean Diet (MD) has been acknowledged as a sustainable diet due to its potential ability to mitigate climate change and its reduced environmental impact on water, soil, and energy use [33]. Encouraging MD adherence might thus serve as an efficient approach to improve our population’s overall health while protecting the country’s environmental assets, as a low adherence to this diet in this population was revealed in a recent study [8].

In our population, there is an obvious reliance on ‘grains and cereals’ as the main contributors to energy intake, and this group contributed significantly to the EFPs in our study. Thus, in addition to encouraging the consumption of whole grains, balancing the EFPs of these items is crucial. This can be done by optimizing irrigation systems in the country, as irrigation management practices significantly influence GHGEs, economic crop water productivity, and soil water content [34].

When it comes to safeguarding the environment, government initiatives like fuel standards upgrades, carbon fees, and laws prohibiting the destruction of old-growth forests have significantly lessened environmental effects than any one person, family, or community could on their own [35]. It is evidenced that a plant-based diet, fewer clothes, and less air travel would only cut greenhouse gas emissions by 25% of what is required to prevent global warming to 1.5 °C over pre-industrial levels, even if everyone on the earth adopted these changes. For instance, many people do not think it is credible to make further “lifestyle” adjustments because they already believe they are doing more to safeguard the environment than governments [35], highlighting the need for effective and efficient policies and public health policies to safeguard the environment and encourage the adoption of more sustainable choices.

### Strengths and limitations

This study assessed the EFPs of the current dietary pattern followed by Lebanese adults. The sample of this study is representative, so the findings, which are valuable as being the most updated findings on this topic in the country, can be generalized for the overall population. In addition, this is the first study in the Arab Region to assess the nitrogen lost to the environment due to consumption, which presents unique and crucial data on a topic that is gaining more attention recently. However, this study has some limitations:, it is important to acknowledge that selecting only one participant per household may have introduced bias by not accounting for within-household heterogeneity. Specific criteria were applied for participant selection, including willingness to participate, availability, and capacity to provide accurate information about dietary habits and household characteristics. Randomization within households was not feasible due to logistical constraints and the voluntary nature of the study. While this approach ensured broad coverage of households and minimized clustering effects, it may limit the exploration of individual differences within larger households. Despite this limitation, the use of stratified cluster sampling and recruitment from diverse channels aimed to capture heterogeneity across households at the community level, enhancing the external validity and generalizability of the findings. In addition, the method used for dietary assessment relies on recalling and is thus subject to inaccuracies (estimating portion sizes or remembering), although facilitating tools were offered to participants to decrease this as much as possible. Moreover, data on nitrogen from production, energy use, and other factors that are taken into consideration when calculating the nitrogen footprint in a country are lacking in Lebanon, so the nitrogen in our study represents one aspect of the footprint that is consumption.

## Conclusion

Lebanon is facing environmental degradation due to many reasons as mentioned earlier. The findings of this study showed that individuals can help in slowing and stopping this destruction by making healthier and more sustainable food choices. Such choices can be easily made by adapting to the Double Pyramid which will eventually lead to better nutritional status and environmental protection. The study findings thus call for public health interventions to allow the adoption of healthier and sustainable food choices. Such interventions can be promoting the adherence to the Double Pyramid while taking into account the population’s preferences and ability to afford healthier and more sustainable choices.

## Supplementary Information

Additional File 1. Table S1. Food Items included in every food group.

Additional File 2. Table S2. EFPs of food items.

## Data Availability

Data is provided within the manuscript or supplementary information files.

## Abbreviations

BMI: Body mass index
Cap/d: Capita per day
CH4: Methane
CI: Crowding index
CO_2_: Carbon dioxide
CO_2_-eq: Carbon dioxide equivalent
EFP: Environmental footprints
ESFC: Environmentally Sustainable Food Consumption
FFQ: Food frequency questionnaire
g/d: Grams per day
GHGE: Greenhouse gas emissions
Kcal: Kilocalories
L/d: Liters per day
MD: Mediterranean Diet
m^3^: Cubic meter
N_2_O: Nitrous oxide
NR-NCDs: Nutrition-related non-communicable diseases
SD: Standard deviation
SDG: Sustainable development goals
UK: United Kingdom
UNDP: United Nations Development Programme
UNEP: United Nations Environment Programme
US: United States
USDA: United States Department of Agriculture
