# Supplementary material for: Greenhouse gas emissions (GHGE), water footprint and nitrogen loss associated with food consumption among adults: findings from the updated LEBANese natiONal food consumption survey (LEBANON-FCS)
Source: BMC Nutr. 2025 Jan 27;11:23. doi: 10.1186/s40795-025-01004-6 (PMC11771036; doi:10.1186/s40795-025-01004-6)
Supplement: Supplementary file 1 — Additional File 1. Table S1. Food Items included in every food group. [file 40795_2025_1004_MOESM1_ESM.docx]

**Additional Files**

**Additional File 1. Table S1.** Food Items included in every food group

| **Food Groups** | **Items** |
| --- | --- |
| Bread, Grains, Cereals and Cereal-Based Products | All kinds of breads, Cereals, rice, rice-based dishes, pasta, oat, bulgur, Lebanese kaake, quinoa |
| Legumes | All kinds of legumes, legume-based dishes, peas and beans, lentils |
| Starchy Vegetables | Potatoes (including potato-based dishes), corn, green beans |
| Vegetables | Raw vegetables (including all kinds of vegetables and salads), cooked vegetables, pickled vegetables, vegetable-based traditional dishes, tomato paste, vegetables soups |
| Olives, Nuts & Seeds | Olives, all kinds of nuts and seeds (including seeds, almonds, cashew nuts, pistachios, walnuts, pine nut, hazelnut, coconut) |
| Dairy Products | All kinds of milk (whole, low fat…) |
|  | All kinds of cheese, yogurt and yogurt-based dishes, laban, kariche, labneh (strained yogurt) |
|  | Milk-based dishes, puddings, frozen and fruit yogurt |
| **Meat, Poultry, Fish, Eggs** |  |
| Red Meat | Meat and organ meats |
| Processed Meat | Processed Meat (hotdog, salami, mortadella, sausages) |
| Poultry | Poultry and poultry organs (chickens, birds) |
| Fish | All kinds of seafood (tuna, sardines, fish, seafood) |
| Eggs | Eggs |
| **Fruits, Total** |  |
| Fruits | All fruits, fruit salads and dried fruits |
| Fresh Fruit Juices | Juices made from 100% fruits |
| **Sweets and Added Sugars** |  |
| Sweets and Snacks | Candies, cakes, pastries, traditional sweets (Jello, custard, Arabic sweets), ice cream, doughnuts, chocolate, biscuits with cream, chips, popcorn, salty biscuits |
| Added Sugars | Added sugars, jams, honey, molasses |
| Hot Beverages | Coffee, tea, Nescafe with/out coffee mate, Infusions |
| Sugar Sweetened Beverages | Commercial and Sweetened juices, regular & light soft drinks, energy drinks |
| Alcoholic Beverages | Spirits and alcohols |
| Added Fats and Oils | Margarine, butter, vegetable oils (palm, sunflower, canola, corn, coconut), animal-based fat |
| Olive Oil | Olive oil |

*****Fast food items (pizza, burgers, manakeesh, fries, etc.) were divided into individual items and classified accordingly.
