# Supplementary material for: Greenhouse gas emissions (GHGE), water footprint and nitrogen loss associated with food consumption among adults: findings from the updated LEBANese natiONal food consumption survey (LEBANON-FCS)
Source: BMC Nutr. 2025 Jan 27;11:23. doi: 10.1186/s40795-025-01004-6 (PMC11771036; doi:10.1186/s40795-025-01004-6)
Supplement: Supplementary file 2 — Additional File 2. Table S2. EFPs of food items. [file 40795_2025_1004_MOESM2_ESM.docx]

**Additional Files**

**Additional File 2. Table S2.** EFPs of food items

| **Food Item** | **GHG (kg CO2-eq)/kg** | **Water Use (L/kg)** | **Nitrogen Factor** |
| --- | --- | --- | --- |
| White Bread | 0.98 | 1,300 | 5.7 |
| Whole Bread | 0.98 | 1,300 | 5.8 |
| Breakfast Cereals | 2.64 | 1,977 | 6.25 |
| Oat | 0.87 | 2,536 | 5.83 |
| Cereal Bar | 0.7 | 771 | 6.25 |
| Rice | 3.17 | 3,400 | 5.95 |
| Bulgur | 1.31 | 1,081 | 5.83 |
| Pasta | 1.98 | 1,390 | 6.25 |
| Potato | 0.58 | 900 | 6.25 |
| Potato Chips | 0.58 | 900 | 6.25 |
| Potato, Fries | 0.58 | 900 | 6.25 |
| Avocado | 0.54 | 1,901 | 6.25 |
| Ketchup | 1.46 | 534 | 6.25 |
| Quinoa | 0.96 | 1,795 | 6.25 |
| Corn, Green Peas | 1.42 | 828.5 | 6.25 |
| Fava Beans | 1 | 5,053 | 6.25 |
| Lentils | 1.03 | 1,001 | 6.25 |
| Chickpeas | 0.67 | 4,177 | 6.25 |
| Milk | 1.13 | 1,000 | 6.38 |
| Yogurt | 1.13 | 1,000 | 6.38 |
| Labneh (Strained Yogurt) | 1.13 | 1,000 | 6.38 |
| Cheese | 8.78 | 5,000 | 6.38 |
| Watermelon | 0.52 | 185 | 6.25 |
| Pear | 0.29 | 922 | 6.25 |
| Plums | 0.27 | 2180 | 6.25 |
| Mango | 0.42 | 1119.56 | 6.25 |
| Pumpkin | 0.38 | 479 | 6.25 |
| Fresh Fruit and Fruit Salad | 0.42 | 600 | 6.25 |
| Dried Fruits | 1.07 | 3,569 | 6.25 |
| Lettuce | 0.4 | 212 | 6.25 |
| Carrot | 0.23 | 195 | 6.25 |
| Seasonal Vegetables | 0.72 | 106 | 6.25 |
| Greenhouse vegetables | 4.42 | 106 | 6.25 |
| Salads (Season - Tabboule - Fattoush) | 0.84 | 708 | 6.25 |
| Chicken | 4.83 | 3,900 | 6.25 |
| Meat | 31.4 | 15,500 | 6.25 |
| Fish | 4.27 | 2,555 | 6.25 |
| Eggs | 5.23 | 3,300 | 6.25 |
| Turkey Ham | 5.11 | 4,325 | 6.25 |
| Organ Meat | 31.4 | 7,746 | 6.25 |
| Bacon | 4.03 | 6,116 | 6.25 |
| Processed Meat (Pork, sausages) | 5.36 | 4,800 | 6.25 |
| Mayonnaise | 4.56 | 4,100 | 6.25 |
| Nuts & Seeds | 0.42 | 8,671.5 | 5.3 |
| Olives | 0.59 | 4,134.23 | 6.25 |
| Coconut | 1.78 | 1,971 | 6.25 |
| Sugar, Honey, Jam, Molasses | 0.47 | 1,500 | 6.25 |
| Chocolate | 3.14 | 10,047 | 6.25 |
| Sweets | 3.7 | 3,140 | 6.25 |
| Biscuits | 2.3 | 1,800 | 5.8 |
| Ice cream | 3.97 | 4,546 | 6.25 |
| Custard | 2.5 | 1,441 | 6.25 |
| Meghli | 0.51 | 636.5 | 6.25 |
| Kounafa | 4.96 | 2,134 | 6.25 |
| Olive Oil | 3.84 | 4,900 | 6.25 |
| Vegetable Oil | 3.89 | 4,900 | 6.25 |
| Vegetable Soup | 0.6 | 1,288.65 | 6.25 |
| Solid Fat (Butter, Ghee) | 8.8 | 5,000 | 6.38 |
| Margarine | 1.43 | 1,324 | 6.25 |
| Tomato Sauce | 1.19 | 151 | 6.25 |
| Soy Sauce | 1.95 | 613 | 6.25 |
| Hot Beverages | 0.49 | 1,040 | 6.25 |
| Soft Drinks (Regular & Light) | 0.43 | 874.9 | 6.25 |
| Spirits & Alcohol | 0.81 | 166 | 6.25 |
| Commercial Fruit Juices | 2.71 | 874.9 | 6.25 |
| Fresh Fruit Juices | 0.48 | 1,026 | 6.25 |
